# Systematic Search for Novel Circulating Biomarkers Associated with Extracellular Vesicles in Alzheimer’s Disease: Combining Literature Screening and Database Mining Approaches

**DOI:** 10.3390/jpm11100946

**Published:** 2021-09-23

**Authors:** David Vogrinc, Katja Goričar, Tanja Kunej, Vita Dolžan

**Affiliations:** 1 Pharmacogenetics Laboratory, Institute of Biochemistry and Molecular Genetics, Faculty of Medicine, University of Ljubljana, 1000 Ljubljana, Slovenia; david.vogrinc@mf.uni-lj.si (D.V.); katja.goricar@mf.uni-lj.si (K.G.); 2 Department of Animal Science, Biotechnical Faculty, University of Ljubljana, 1000 Ljubljana, Slovenia

**Keywords:** Alzheimer’s disease, biomarker, miRNA, extracellular vesicles

## Abstract

miRNAs play an important role in neurodegenerative diseases. Many miRNA-target gene interactions (MTI) have been experimentally confirmed and associated with Alzheimer’s disease (AD). miRNAs may also be contained within extracellular vesicles (EVs), mediators of cellular communication and a potential source of circulating biomarkers in body fluids. Therefore, EV-associated miRNAs (EV-miRNAs) in peripheral blood could support earlier and less invasive AD diagnostics. We aimed to prioritize EV-related miRNA with AD-related genes and to identify the most promising candidates for novel AD biomarkers. A list of unique EV-miRNAs from the literature was combined with a known set of AD risk genes and enriched for MTI. Additionally, miRNAs associated with the AD phenotype were combined with all known target genes in MTI enrichment. Expression in different sample types was analyzed to identify AD-associated miRNAs with the greatest potential as AD circulating biomarkers. Four common MTI were observed between EV-miRNAs and AD-associated miRNAs: hsa-miR-375–*APH1B*, hsa-miR-107–*CDC42SE2*, hsa-miR-375–*CELF2*, and hsa-miR-107–*IL6*. An additional 61 out of 169 unique miRNAs (36.1%) and seven out of 84 unique MTI (8.3%), observed in the body fluids of AD patients, were proposed as very strong AD-circulating biomarker candidates. Our analysis summarized several potential novel AD biomarkers, but further studies are needed to evaluate their potential in clinical practice.

## 1. Introduction

Progressive neurodegeneration is a feature of various age-related brain disorders, including Alzheimer’s disease (AD). AD is the leading cause of dementia, and the increase in lifespan is making it one of the most important global health issues. Mild cognitive impairment (MCI) is the main feature of the pre-dementia stage of the disease, while severe memory and learning dysfunction can be observed during disease progression [1]. Typical AD cases are also known as late-onset AD. Onset of symptoms before 65 years of age is uncommon and is regarded as early-onset AD [2]. Multiple risk factors contribute to the development of AD and the mechanisms of disease pathogenesis are still not completely understood [3].

Various genetic risk loci contribute to the development of the disease. For the most frequent form of the disease, sporadic AD, there are no common causative genes. Different studies report that rather than single genes, polygenic risk scores can be used to predict AD risk [4,5,6]. Numerous GWAS have identified AD risk loci [7,8,9]. Apart from genetic background, epigenetic mechanisms may play an important role in AD pathogenesis as well [10]. Small, non-coding RNA have been extensively studied in neurodegenerative diseases. miRNAs are involved in post-transcriptional regulation of gene expression. Upon binding primarily to the 3′ untranslated region of the messenger RNAs (mRNAs), miRNAs block the translation or lead to degradation of target mRNAs [11]. One miRNA can target multiple mRNAs, and many interactions have been associated with disease mechanisms [12]. More than 380,000 experimentally validated miRNA-target gene interactions (MTI) have been reported in *H. sapiens* alone [13]. Data on MTI are important as they may improve our understanding of metabolic processes and biological pathways associated with neurodegenerative changes in the brain. 

Although AD diagnostic criteria are well established, lack of specificity and sensitivity in AD diagnoses can be observed [14]. Since there is no currently available treatment for the advanced state of the disease, early diagnostics of preclinical AD is extremely important. Detectable cerebrospinal fluid (CSF) proteins reflect cerebral accumulation of insoluble plaques and aggregation of neurofibrillary tangles (NFT), two major hallmarks of AD [15]. Decreased amyloid-β (Aβ_1–42_), increased total Tau (tTau), and phospho Tau (pTau) from CSF are used in clinical practice as suitable biomarkers to support AD diagnostics [16]. Measurement of CSF Aβ_40_, Aβ_42_, pTau, and tTau showed good diagnostic accuracy in discriminating AD patients from non-AD patients, with the Aβ_42/40_ ratio performing best [17,18,19,20]. However, accumulation of protein aggregates is common between different neurological disorders, especially in early stages of the disease [21,22,23]. Discrepancies in CSF biomarker measurement approaches between different clinical centers add to the variability in AD diagnostics. Hence, studies are trying to identify novel reliable AD-specific biomarkers capable of sensing initial neurodegenerative changes in the brain. Circulating miRNAs and target genes bear great potential as AD-related biomarkers and could provide valuable insight into the cellular mechanisms of AD pathology [24].

Extracellular vesicles (EVs) are being extensively studied as source of novel AD-related biomarkers. EVs play a key role in intercellular communication as they harbor proteins, RNAs, and lipids with important functions in the central nervous system [25]. Whether EVs contribute to pathophysiological changes observed in AD is still subjected to discussion. Both amyloid and tau pathologies have been associated with neuronal-derived EVs. Release of Aβ from cells in vitro and Aβ-induced synaptic disruption in vivo was associated with EVs [26,27,28]. AD-associated tau phosphoforms have been found in EVs isolated from AD patients [29]. Furthermore, brain-derived EVs are able to cross the blood–brain barrier, suggesting they could have potential as circulating biomarkers in body fluids [30]. EV cargo has been extensively studied in neurodegenerative disorders. Differentially expressed EVs enriched mRNAs and miRNAs in blood serum and CSF were reported (reviewed in [31]). Several case-control studies identified miRNAs enriched in EVs (EV-miRNAs) isolated from body fluids in AD patients [24,32,33,34,35]. These findings suggest EV-miRNAs may have an important role in complex regulatory networks of AD. Better understanding of miRNA function in AD pathogenesis could eventually contribute to the development of novel earlier and less invasive diagnostic approaches. 

Therefore, the aim of our study was to prioritize EV-related miRNA biomarkers of AD based on interactions with known AD-related genes. Additionally, we wanted to identify the most promising circulating miRNAs and their MTI that could serve as biomarkers in AD.

## 2. Methods

Two different approaches, based on literature screening or database mining, were used in search for miRNA-target interactions in Alzheimer’s disease. 

**Figure 1:**
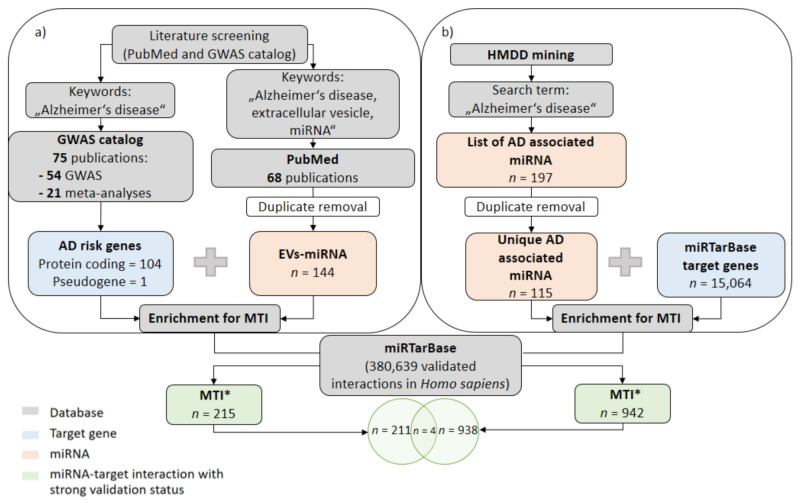
Integration of literature screening and database mining for AD-related miRNAs and target gene evaluation and prioritization of MTI: (**a**) Previously published work on AD risk genes was combined with a list of miRNAs associated with extracellular vesicles obtained from literature search. miRNA-target interactions on various phenotypes and diseases were extracted from miRTarBase. (**b**) Apart from that, HMDD mining resulted in list of AD-associated miRNAs that were combined with the complete list of genes in miRTarBase for evaluation of miRNA-target interactions. AD = Alzheimer‘s disease; EVs = extracellular vesicles; HMDD = Human microRNA Disease Database; MTI = miRNA-target interaction.

In the first approach, we tried to identify MTI with known AD-related genes for miRNAs enriched in EVs in AD. A PubMed search for original articles published from November 2014 until the end of February 2021 was performed using keywords “Alzheimer‘s disease, extracellular vesicle, miRNA”. A list of unique EV-miRNAs was combined with a known set of genes previously associated with AD risk and biomarker levels in GWAS studies [36]. Enrichment for MTI was performed by using miRTarBase (http://mirtarbase.cuhk.edu.cn/php/index.php, accessed on 25 March 2021) (Figure 1a). In miRTarBase, experimentally validated MTI are reported according to different confirmation methods [13]. 

In the second approach, we tried to identify MTI for all miRNAs associated with AD reported in the Human microRNA Disease Database—HMDD (https://www.cuilab.cn/hmdd, accessed on 25 March 2021). The HMDD annotates miRNAs associated with a specific disease phenotype [12]. After duplicate removal, a list of all unique AD-associated miRNAs was combined with all known target genes in miRTarBase for MTI enrichment (Figure 1b). 

**Figure 2:**
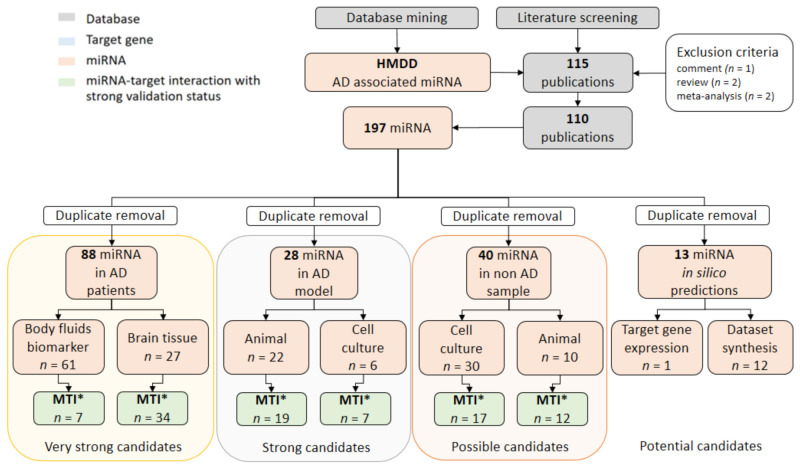
Data extraction of AD-associated miRNAs from HMDD and manual literature curation of experimentally validated MTI. Database mining resulted in a list of publications, investigating the role of derived miRNAs that were manually screened for their abundance in different sample types. miRNAs were curated as very strong candidates (association observed in a sample of AD patients), strong candidates (association observed in AD cell or animal models), and possible candidates (association observed in non-AD cell or animal models). In silico predictions were also highlighted as potential AD-associated candidates, suggesting further research. AD = Alzheimer‘s disease; HMDD = Human microRNA Disease Database; MTI* = miRNA-target interaction, extraction from previously published literature.

Furthermore, a list of all AD-associated miRNAs from HMDD was prioritized according to the level of evidence. In a search for miRNAs with the greatest potential as AD circulating biomarkers, their expression in different sample types was reviewed (Figure 2). As reported in the original literature, candidate miRNAs were observed in biological samples of AD patients (very strong candidates), AD animal or cell culture models (strong candidates), non-AD animal or cell culture models (possible candidates), and in silico predictions (potential candidates). Original papers were screened for reported MTI. Overlap between different categories was visualized with a Venn diagram using the Venny tool [37].

## 3. Results

Based on the literature search, 144 unique EV-miRNAs were found (Figure 1a). In the HMDD, 115 unique AD-associated miRNAs were reported (Figure 1b). Only six miRNAs were common between both datasets: hsa-miR-29c, hsa-miR-136-3p, hsa-miR-16-2, hsa-miR-132-5, hsa-miR-331-5p, and hsa-miR-485-5p. Combining EV-miRNAs from 68 publications with a list of 105 AD-related genes resulted in 215 specific MTI. HMDD screening resulted in 942 MTI. An overlap between the two approaches showed four common MTI, hsa-miR-375–*APH1B*, hsa-miR-107–*CDC42SE2*, hsa-miR-375–*CELF2*, and hsa-miR-107–*IL6,* with the greatest potential as EV-miRNA circulating biomarkers (Table 1).

**Figure 3:**
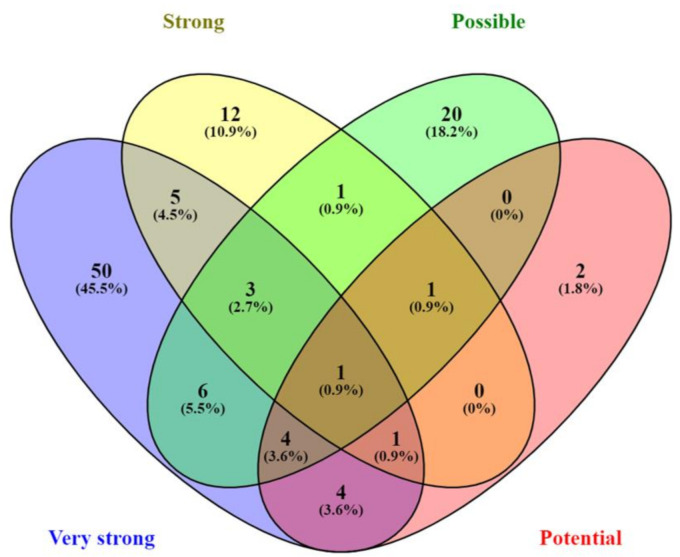
Overlap of common identified miRNAs between different candidate categories, according to the level of evidence.

After duplicate removal, prioritization of 169 unique HMDD miRNAs associated with AD in different sample types resulted in 88 very strong AD biomarker candidates (Figure 2, Table S1). While 61 (36.1%) of them served as body fluids biomarker, 27 (16%) were differentially expressed in brain tissue. Only 14 miRNAs were observed both in body fluids and brain samples. A total of 28 miRNAs regarded as strong candidates were observed in animal (22) or cell culture (6) models of AD. Only four miRNAs were common between both models. Expression of another 40 possible candidates was observed in other, non-AD cell culture (30) or animal models (10), with an overlap of four miRNAs. On the other hand, 13 miRNAs were linked to AD by in silico predictions. The overlap between different prioritization categories can be seen in a Venn diagram in Figure 3. Only one miRNA, hsa-mir-34a, was observed in all four categories.

For all HMDD-identified miRNAs, we proposed key AD-related MTI by screening original papers (Figure 2, Table S1). We identified seven (8.3%) experimentally validated MTI from 84 unique MTI in the body fluids of AD patients, while 34 (40.5%) MTI were confirmed in AD brain tissue samples. Another 19 MTI were found in AD animal models and seven were associated with AD cell cultures. In non-AD samples, 17 MTI were found in cell cultures and 12 in animal models. Seven MTI prioritized to have the greatest potential as novel AD biomarkers are highlighted in Table 2.

## 4. Discussion

miRNAs and their target genes are extensively studied as potential AD circulating biomarkers. We prioritized EV-related miRNA biomarkers interacting with known AD-related genes and identified the most promising circulating miRNAs and their MTI that could serve as biomarkers for AD.

Four EV-associated MTI (hsa-miR-375–*APH1B*, hsa-miR-107–*CDC42SE2*, hsa-miR-375–*CELF2*, and hsa-miR-107–*IL6*) were identified by our combined literature screening and database mining approaches. Using the HMDD, seven MTI (hsa-miR-193b–*APP*, hsa-miR-29c–*BACE1*, hsa-miR-613–*BDNF*, hsa-miR-29c–*DNMT3*, hsa-miR-206–*IGF1*, hsa-miR-128–*PPARG*, and hsa-miR-146a–*TLR2*) were observed in the body fluids of AD patients. 

Two miRNAs were prioritized as the most promising EV-related biomarkers based on interactions with known AD-related genes: hsa-miR-375 and hsa-miR-107. Microarray analysis identified two EV-related hsa-miR-375 target genes, *APH1B* and *CELF2*. *APH1B* encodes for anterior pharynx defective-1 protein, a crucial part of the γ-secretase complex. Together with PS1/PS2 and PEN2, it is involved in cleavage of amyloid-precursor proteins (APP) in the amyloid cascade [48]. Depletion of *Aph1b* in mice leads to a progressive neurodegenerative phenotype and indicates *APH1B* as a potential AD treatment target [49]. APP, PS1, and PS2, encoded by *APP*, *PSEN1*, and *PSEN2*, are common causative genes for a familial, early-onset type of AD [2]. Therefore, *APH1B* was also proposed as an AD-risk locus. *APH1B* missense variant rs117618017 was recently identified as a high-confidence AD risk variant [8,50]. CELF2 is an RNA-binding protein implicated in alternative splicing of TREM2 [51]. The effect of TREM2 in neuronal inflammation, present also in AD, has been extensively studied. TREM2 is a key player in the microglial response to increased amyloid burden [52]. Multiple genetics studies revealed *TREM2* as an important AD risk locus [9,53,54]. Furthermore, *CELF2* rs201119 was associated with increased AD risk [55]. Interaction of hsa-miR-375 with both *APH1B* and *CELF2* was experimentally confirmed in a gastric carcinoma sample [38]. The importance of hsa-miR-375 has also been studied in neurological disorders. The effect of hsa-miR-375 in promoting oxidative stress accompanying AD has been proposed [56]. Furthermore, hsa-miR-375 was differentially expressed in CSF and was highlighted as an AD biomarker [24]. 

Two EV-related hsa-miR-107 target genes, *CDC42SE2* and *IL6*, were confirmed using different experimental approaches. *CDC42SE2* is a potential actin cytoskeleton modulator acting downstream of *CDC42* [57]. The importance of actin in AD pathology and involvement in synaptotoxicity has previously been established [58,59]. *CDC42SE2* rs382216 was associated with decreased AD risk in a GWAS study [60]. Cytokines are generally recognized as important mediators of inflammation. Multiple lines of evidence link IL6 with neurodegeneration. Elevated IL6 levels have been observed in AD brain tissue [61,62]. IL6 is also important in neuronal cell growth and differentiation [63]. Recently, rs1800796 in *IL6* has been associated with increased AD risk in a meta-analysis [64]. Furthermore, multiple polymorphisms in gene coding for IL6 receptors (*IL6R*) were found in AD GWAS studies [65,66]. Thus, the importance of both *IL6* and *IL6R* in the genetic predisposition of disease imply the importance of cytokines in the development of AD. Experimental validation of hsa-miR-107 interactions with *CDC42SE2* and *IL6* were already reported [39,40]. In an AD cell model, a decrease in hsa-miR-107 level was observed, suggesting its function in disease progression [67]. The potential of hsa-miR-107 as an AD circulating biomarker was proposed, since lower expression in blood plasma was observed in AD patients [68]. 

Six miRNAs were prioritized as the most promising circulating biomarkers based on HMDD data. Seven MTI prioritized in the database mining approach were confirmed with reliable methods—reporter assay, RT-qPCR, and Western blot. One MTI, hsa-miR-193–*APP*, was also observed in EVs [41]. 

Multiple miRNA target genes were associated with metabolic processes and regulation of gene expression. The interaction of exosomal hsa-miR-193b with *APP* was experimentally confirmed in an AD mouse model and a sample of AD patients [41]. Later on, the potential of hsa-miR-193b as an early diagnostic circulating biomarker was confirmed [69,70]. *APP* encodes for amyloid precursor proteins and is the initial part of the amyloidogenic pathway [15]. APP is involved in synapse formation and stability and thus highly enriched in brain tissue [71]. Another gene linked to synaptic function is *BACE1*. BACE1 is a secretase, mediating a two-step generation of Aβ through APP cleavage [72]. Since it has been discovered in CSF, the potential of BACE1 as an AD biomarker was extensively studied [73,74,75,76,77]. *BACE1* polymorphisms were also associated with AD [78,79]. Interaction of hsa-miR-29c with both *BACE1* and *DNMT3* was experimentally confirmed in CSF or blood samples [42,44]. De novo methyltransferase DNMT3 established methylation patterns on DNA and had an important role in genome imprinting [80]. Potential use of hsa-miR-29c as an AD circulating biomarker was further confirmed using RNA deep sequencing [81]. Brain-derived neurotrophic factor (BDNF) is another important protein involved in AD pathology. BDNF mediates survival and differentiation of neurons [82]. In AD brains, decreased BDNF levels have been reported [83]. In addition, BDNF decreases in serum during the initial stages of AD, suggesting BDNF has an important function in early synaptic dysfunctions [84]. Furthermore, *BDNF* polymorphisms have been associated with AD [85,86]. hsa-miR-613 as an interacting partner of *BDNF* was detected in the serum and CSF of AD patients and AD mouse models [43]. 

One miRNA target gene was involved in immune response, an important companion of AD. The function of hsa-miR-146a in AD has been extensively studied. Recently, hsa-miR-146a upregulation was observed in postmortem AD brain tissue [87]. The effect of hsa-miR-146a as a switch for reduced proinflammatory microglial phenotypes was proposed [88]. Increased AD risk effect of rs2910164 minor allele in the miR-146a coding region was reported [47,89]. Multiple studies proposed hsa-miR-146a as an AD circulating biomarker [81,90,91]. An experimental interaction of hsa-miR-146a with *TLR2* was reported [47]. Toll-like receptor 2, encoded by *TLR2*, is a key component in the innate immune system. TLR2 are one of the most studied pattern recognition receptors that recognize pathogens and initiate the cascade of host defense mechanisms [92]. The activation of TLR2 induces neurodegeneration and cognitive deficit in AD murine models [93,94]. Further evidence elucidated the role of immune system in AD development, including *TLR2* polymorphisms associated with AD in Asian populations [95,96].

Another two target genes were associated with glucose metabolism. PPARG is a receptor involved in lipid and glucose metabolism [97]. Interaction of a neuroprotective agent with PPARG has been discussed, suggesting the importance of *PPARG* in Aβ formation during inflammation [98]. In addition, the effect of *PPARG* polymorphisms on AD risk and age of onset was evaluated [99,100]. Upregulation of hsa-miR-128 lead to downregulation of *PPARG* in clinical AD samples and AD cell models [46]. Furthermore, the importance of hsa-miR-128 in AD pathology suggests impaired amyloid clearance associated with hsa-miR-128 upregulation and reduced Aβ production in miR-128 knock-out mice [101,102]. Recently, hsa-miR-128 was proposed as potential AD circulating biomarker [103]. Insulin growth factor-1 (IGF1) is a hormonal regulator of insulin resistance in diabetes. The effects of IGF1 on cell survival, apoptosis, and stimulation of neurogenesis in the hippocampus can predict neurodegeneration, encompassing AD [104]. Decreases in serum levels of IGF1 have been linked to AD [105]. The effect of diabetes as an AD risk factor has also been extensively studied. IGF1 levels were associated with cognitive decline in diabetic patients [106]. In terms of genetics, only one *IGF1* polymorphism was associated with AD risk in the Chinese Han population [107]. Upregulation of hsa-miR-206 in AD blood samples was linked to *IGF1* and AD-related inflammation [45]. Further studies confirmed the potential of hsa-miR-206 as a circulating AD biomarker [69,108,109]. Furthermore, hsa-miR-206 was proposed as a novel AD pharmacological target [110].

In a search for novel, reliable AD circulating biomarkers from publicly available databases, we determined the most promising AD-related miRNA candidates and their MTI using two different approaches. The overlap between two comprehensive miRNA datasets resulted in only a few common MTI. However, not all specific MTI were confirmed in an AD sample. Contrary to our expectation, the EV-associated miRNA list did not overlap with the extensive list of miRNAs linked to AD phenotype. To better understand the function of enriched MTI in AD, a manual curation according to previously published evidence was performed. We observed no overlap between MTI obtained from EV-miRNA enrichment and very strong candidate MTI from manual prioritization. However, not all studies confirmed that miRNAs identified in our study can serve as suitable biomarkers for AD. For example, even though they were often associated with AD, no significant differences in expression levels of miR-146a, miR-107, miR-375, and miR-29c in CSF or blood samples were observed in some studies [111,112,113,114,115,116,117]. In addition, statistical analysis of candidate biomarkers could help in prioritization of miRNAs as AD biomarkers. Further studies are therefore needed to confirm the role of these miRNAs and their MTI in AD.

Although hsa-mir-34a, hsa-miR-136-3p, hsa-miR-16-2, hsa-miR-132-5, hsa-miR-331-5p, and hsa-miR-485-5p were proposed as AD biomarkers using both approaches, no significant MTI were identified. The majority of included studies reported miRNA expression as up- or downregulated, normalized to other common miRNAs. Determination of cut-off values for miRNA expression is challenging and rarely reported [70,118]. In the design of future studies, this should also be taken into account. Additionally, in body fluids, miRNAs are abundant as cell free or enriched in EVs. As the potential of neuronal-derived EVs in peripheral blood has been extensively studied, we focused on EVs as a promising source of miRNAs. EV-enriched miRNAs set can differ from total miRNAs in a sample, which was also highlighted by our analysis. Selection of appropriate isolation methods based on different biomarker types is therefore needed for reliable biomarker detection. Our results therefore suggest that a combination of different publicly available miRNA databases should be evaluated in a search for the most promising biomarker candidates associated with AD. However, miRNAs and target genes, highlighted under two different approaches, can be regarded as top novel circulating biomarker candidates in AD. Additionally, the potential of very strong candidates identified in the HMDD as AD circulating biomarkers should be further researched, also in the EVs of body fluids.

## 5. Conclusions

In conclusion, in the present study we prioritized several important experimentally confirmed interactions between miRNAs and target genes implicated in AD. miRNAs hsa-miR-193b, hsa-miR-29c, hsa-miR-613, hsa-miR-206, hsa-miR-128, and hsa-miR-146a represent the most promising circulating AD biomarkers, while miRNAs hsa-miR-375 and hsa-miR-107 could be promising EV-related AD biomarkers. Further studies are needed to evaluate the potential of key identified miRNAs in clinical practice. Elucidating the complex network of AD-related miRNAs and target genes could eventually also enable identification of novel therapeutic targets for AD.

## Figures and Tables

**Table 1:** Common experimentally confirmed MTI identified by both literature screening and database mining approaches.

miRNA	Target Gene	Method	PubMed ID (Reference)
hsa-miR-375	*APH1B*	Microarray	20215506 [38]
hsa-miR-375	*CELF2*	Microarray	20215506 [38]
hsa-miR-107	*CDC42SE2*	PAR-CLIP ^1^	21572407 [39]
hsa-miR-107	*IL6*	Luciferase reporter assay, RT-qPCR ^2^, Western blot	24429361 [40]

^1^ PAR-CLIP: photoactivatable ribonucleoside-enhanced crosslinking and immunoprecipitation; ^2^ RT-qPCR: reverse transcription quantitative polymerase chain reaction.

**Table 2:** Experimentally confirmed MTI prioritized as very strong AD-related candidates.

miRNA	Target Gene	Method	PubMed ID (Reference)
hsa-miR-193b	*APP*	Luciferase reporter assay, RT-qPCR ^1^, Western blot	25119742 [41]
hsa-miR-29c	*BACE1*	Luciferase reporter assay, RT-qPCR ^1^, Western blot	25955795 [42]
hsa-miR-613	*BDNF*	EGFP reporter assay, RT-qPCR ^1^, Western blot	27545218 [43]
hsa-miR-29c	*DNMT3*	Luciferase reporter assay, RT-qPCR ^1^, Western blot	25815896 [44]
hsa-miR-206	*IGF1*	Luciferase reporter assay, RT-qPCR ^1^, Western blot	27277332 [45]
hsa-miR-128	*PPARG*	Luciferase reporter assay, RT-qPCR ^1^, Western blot	30328325 [46]
hsa-miR-146a	*TLR2*	Luciferase reporter assay, RT-qPCR ^1^, Western blot	26095531 [47]

^1^ RT-qPCR: reverse transcription quantitative polymerase chain reaction.

## Data Availability

For preparation of the manuscript, the following publicly available databases were used: miRTarBase (https://mirtarbase.cuhk.edu.cn/~miRTarBase/miRTarBase_2019/php/index.php, accessed on 25 March 2021); the HMDD (https://www.cuilab.cn/hmdd, accessed on 25 March 2021). All the data are presented within the article and in the supplementary material.

## Supplementary Materials

The following are available online at https://www.mdpi.com/article/10.3390/jpm11100946/s1, Table S1: List of all AD-associated miRNAs from the HMDD database.
